# A Direct Compression Matrix Made from Xanthan Gum and Low Molecular Weight Chitosan Designed to Improve Compressibility in Controlled Release Tablets

**DOI:** 10.3390/pharmaceutics11110603

**Published:** 2019-11-12

**Authors:** Deeb Abu Fara, Suha M. Dadou, Iyad Rashid, Riman Al-Obeidi, Milan D. Antonijevic, Babur Z. Chowdhry, Adnan Badwan

**Affiliations:** 1 Chemical Engineering Department, School of Engineering, University of Jordan, Amman 11942, Jordan; 2 Department of Science, Faculty of Engineering & Science, University of Greenwich, Medway Campus, Chatham Maritime, Kent ME4 4TB, UK; S.Dadou@qub.ac.uk (S.M.D.); M.Antonijevic@greenwich.ac.uk (M.D.A.); b.z.chowdhry@greenwich.ac.uk (B.Z.C.); 3 Research and Innovation Centre, The Jordanian Pharmaceutical Manufacturing Company (JPM), P.O. Box 94, Naor 11710, Jordan; irashid@jpm.com.jo (I.R.); riman.obeidi4@gmail.com (R.A.-O.); jpm@go.com.jo (A.B.)

**Keywords:** direct compression, compressibility, compactibility, controlled drug release, Gamlen tablet press, low molecular weight chitosan, tableting, xanthan gum

## Abstract

The subject of our research is the optimization of direct compression (DC), controlled release drug matrices comprising chitosan/xanthan gum. The foregoing is considered from two main perspectives; the use of low molecular weight chitosan (LCS) with xanthan gum (XG) and the determination of important attributes for direct compression of the mixtures of the two polymers. Powder flow, deformation behaviour, and work of compression parameters were used to characterize powder and tableting properties. Compression pressure and LCS content within the matrix were investigated for their influence on the crushing strength of the tablets produced. Response surface methodology (RSM) was applied to determine the optimum parameters required for DC of the matrices investigated. Results confirm the positive contribution of LCS in enhancing powder compressibility and crushing strength of the resultant compacts. Compactibility of the XG/LCS mixtures was found to be more sensitive to applied compression pressure than LCS content. LCS can be added at concentrations as low as 15% *w*/*w* to achieve hard compacts, as indicated by the RSM results. The introduction of the plasticity factor, using LCS, to the fragmenting material XG was the main reason for the high volume reduction and reduced porosity of the polymer mixture. Combinations of XG with other commonly utilized polymers in controlled release studies such as glucosamine, hydroxypropyl methylcellulose (HPMC), Na alginate (ALG), guar gum, lactose and high molecular weight (HMW) chitosan were also used; all the foregoing polymers failed to reduce the matrix porosity beyond a certain compression pressure. Application of the LCS/XG mixture, at its optimum composition, for the controlled release of two model drugs (metoprolol succinate and dyphylline) was examined. The XG/LCS matrix at 15% *w*/*w* LCS content was found to control the release of metoprolol succinate and dyphylline. The former preparation confirmed the strong influence of compression pressure on changing the drug release profile. The latter preparation showed the ability of XG/LCS to extend the drug release at a fixed rate for 12 h of dissolution time after which the release became slightly slower.

## 1. Introduction

Tableting, in pharmaceutical applications, has traditionally been carried out by using direct compression (DC) processing due to its simplicity, environmentally friendly (solvent/heat-free) nature and time/cost-effectiveness [1]. It is also considered suitable for large-scale, continuous production of pharmaceutical products [2]. DC is the preferred tablet preparation method when the mixed powders of the active pharmaceutical ingredients (APIs) and excipients are physically compressible and stable under high compression forces. In addition, DC processing enhances the stability of APIs since no aqueous treatment is needed during formulation [3,4]. Product development on a DC platform—including immediate and controlled release tablet manufacturing—shows a pronounced reliance on the type of excipients used.

Hydrophilic polymers have been used in controlled release preparations for the last 50 years [5,6,7]. A diverse range of numerous polymers, varying in their origin (natural, semi-synthetic, and synthetic) and mechanism of action have been used in sustained release dosage forms. Naturally occurring xanthan gum (XG) [8] is a widely used polymer for pharmaceutical applications. XG is a hetero, branched, and negatively charged hydrophilic polymer able to swell and/or to form hydrogels in aqueous media [9,10]. In this regard, XG has been extensively investigated for controlled release drug tablet preparations using either wet granulation or direct compression [11]. The former technique is suitable for producing strong compacts, especially because XG in solution is well known to be a strong binding agent [12,13]. The later technique is desired in tableting due to the advantages it imparts with respect to time and cost. However, XG is associated with a number of physical properties that limit its use in DC [14]. For example, it has been found that XG can only produce hard compacts when its concentration exceeds 30%–40% *w*/*w* within the tablet matrix [15,16]. Such a high content of XG has been found to result in undesired/excessive retardation of the release of certain drugs [17]. On the other hand, the use of a low XG content (10%–20% *w*/*w*) in tablets gives rise to low mechanical strength and a tendency to undergo the “burst effect” due to the erosion of XG in an acidic environment, thus resulting in faster drug release [18,19]. The apparent binding deficiencies between the dry XG granules in addition to their low intrinsic porous structure are responsible for the weak, low compressible compacts produced when powders are subjected to compression [20].

When compression enhancers or drug release modifiers were considered in tablet processing for controlled release systems, XG mass content and the strength of compacts used presented the two major attributes for optimized drug release. For example, polyethylene glycol (PEG) was found to enhance drug release via a zero order profile when a high XG mass content was used. However, the crushing strength of such compacts was reduced to values that limit the use of the matrix in DC [21]. The same conclusion was deduced when ionic modifiers containing calcium salts e.g., CaHPO_4_ were used despite the fact that they produce zero order release [22]. In the same context, XG had to be present at a mass content >65% *w*/*w* in order to produce readily compressible and compactable tablets [23].

Addition of polymeric modifiers is the most convenient method used in DC; this is due to the high solid-solid binding capabilities they provide at low mass content within the tablet. The combination of ethyl-cellulose [24] and hydroxypropyl methyl cellulose [25] with XG showed greater drug retardation when used at low content as a copolymer matrix than when XG was used alone. Drug release profiles using these matrices followed a zero order kinetic profile attributed to the porous structure of these polymers or to polymeric modifiers when a gel layer of XG is formed. Furthermore, locust bean gum (LBG) has been added to XG in commercial products (TIMERx^®^) in order to overcome the low compressibility and burst effect of XG in a controlled release matrix that displayed zero order kinetics for the release of metoprolol tartrate [26]. However, a high content of LBG was needed (XG:LBG mass ratio of 1:1) in the matrix preparations. Moreover, drug release profiles were compared at very similar tablet crushing strengths. This implies that a specific controlled release profile is attained at a predetermined matrix porosity. In fact, controlled release matrices at different XG:LBG mass ratios attained similar drug release profiles when they all had the same crushing strength. However, there was no indication of any relationship between the dependence of drug release on tablet porosity when it is changed by varying the compression pressure [26].

Recently, the use of high molecular weight (HMW) chitosan with XG has been used as an effective controlled release system with improved mechanical and flow properties at an XG:chitosan mass ratio of 1:1. Unlike most controlled release matrices, the XG:HMW chitosan matrix involved a simple preparation method via physical mixing. In addition, the matrix needed no further additives i.e., excipients to improve powder flow, compression and compaction properties [27]. Some of these features have been attributed to the physical nature of HMW chitosan. For example, it has been reported that the increase in crushing strength of compacts made of XG:HMW chitosan is attributed to the increase in their porous structure relative to that of compacts made of XG [28,29]. In fact, it is the low compressibility of the foregoing that imparts less bridging contacts between surfaces upon punch displacement when a compression force is applied. In other words, HMW chitosan acts as a porosity enhancing agent. Moreover, compacts composed of XG when used as a single excipient for controlled drug delivery are of weak mechanical strength and consequently of faster drug release. The foregoing was reported for the controlled drug release of a diclofenac sodium preparation containing XG. In this regard, the weak matrix formed accounted for the burst effect during dissolution [18].

The use of porosity modifiers comprising high HMW chitosan has been given a great deal of attention for compression and controlled drug release purposes [27,28,30]. However, as mentioned earlier, the content of HMW chitosan was almost equivalent to the amount of XG within the matrix. In contrast, the choice of low molecular weight chitosan (LCS) was based on previous evidence on its inherent high powder compressibility and compactibility as well as its optimum molecular interaction with XG [30,31].

However, in most reported studies on controlled release tablets, the main focus of research has been on controlling the drug release for a certain time and in a predictable manner, irrespective of compression process attributes [30]. Consequently, investigation of the behaviour of powders comprising controlled release matrices during processing was not of primary interest. This is clear from the fact that the effect of compression parameters was not investigated for optimum tablet processing and, concurrently, for controlled drug release profiles when an XG:LCS matrix was used. In fact, the %LCS content was found to be a major process attribute in order to attain the desired drug release profile. In this context, an LCS content of 15% *w*/*w* provided the most suitable ratio for interaction with XG [32]. However, there is a lack of information as to why LCS content is crucial for enhancing mechanical strength of compacts compared to compression pressure. Ideally a low LCS content is most favourable for increased matrix compressibility, though it is not favourable with regard to the resultant decrease in the mechanical strength of tablets produced. This decrease is attributed to the increased XG content within the XG/LCS matrix [31]. Therefore, compression pressure is an important consideration for optimum tabletability of the matrix since it has a pronounced influence on the crushing strength of tablets.

Thus, the core of the current study is to establish an optimum controlled release formulation using physical mixtures of tablet matrix components based upon an understanding of powder and tableting properties of LCS, XG, and mixtures thereof, during compression. The compression pressure, as a key attribute of these properties, was tested for its role in influencing drug release, alongside the content of controlled release excipient(s). Optimum tableting parameters have been investigated for DC processing of the matrix. Applications of the matrix in controlled release preparations were then examined using metoprolol succinate and dyphylline as model drugs.

## 2. Materials and Methods

### 2.1. Materials

LCS (Batch No. GC20140503) with a viscosity of 11 mPa (1% *w/v* in water), average molecular weight of 13 kDa, degree of deacetylation >90% and particle size of ~100 µm (95% of the sample <100 µm). High molecular weight chitosan with an average molecular weight of 150 kDa, average particle size of 180–100 μm, viscosity of 150 mPas (1% *w/v* in water), and degree of deacetylation of >90% (batch No. GC20140510) and d-glucosamine HCl were all obtained from G.T.C. Union Group LTD., Qingdao, China. XG was purchased from Jungbunzlauer Ladenburg GmbH, Ladenburg, Germany (Batch No. 2504519) having a viscosity range of 1400–1600 mPa (1% *w/v* in water), average molecular weight of 1200 kDa and average particle size less than 180 μm.

Dyphylline, 7-(2,3-dihydroxy-propyl theophylline), 99%, was obtained from ACROS Organics, Geel, Belgium. USP grade metoprolol succinate (MS) was obtained from Ipca Laboratories Ltd., Mumbai, India (batch No. A0337986). Hydroxypropyl methylcellulose (HPMC) was purchased from Dow Chemical Co., Plaquemine, LA, USA. Sodium alginate and guar gum were obtained from Central Drug House, New Delhi, India; α-lactose monohydrate (Pharmatose 200 M) was supplied by DFE Pharma, Veghel, the Netherlands. All other reagents used were of analytical grade and were accessed from the Jordanian Pharmaceutical Manufacturing Co., (JPM), Naor, Jordan.

### 2.2. Experimental Design Using Response Surface Methodology (RSM)

RSM was used to carry out process optimization by employing two input variables representing LCS content and compression pressure and one response variable representing the tablet crushing strength. The first input variable was chosen based on previously reported data using molecular dynamic simulations, whereby the most favourable complex was formed at an LCS content of 15% *w*/*w* within an XG/LCS matrix. Tablet crushing strength, not drug release, was chosen as the response of the current design. The matrix:drug ratio was fixed at a previously reported value of 2.61:1. The foregoing represents the recommended ratio which provides optimum drug release performance [32]. Therefore, in order to attain optimum tabletability, the RSM data were used to determine the range of compression pressures and/or the LCS content that can overcome the low matrix compactibility.

RSM analysis was carried out using Design-Expert^®^ software version 11; the program Central Composite Design was used to build a model employing quadratic parameters for three levels of each factor. These levels were lower and upper limits, in addition to middle values, coded as (−1), (1), and (0), respectively. The factors considered were compression pressure and LCS content; their corresponding codes are listed in Table 1. In the same Table, the star point, or α, representing the extreme axial run, was found to have a value of 1.414 for a two factor design according to Equation (1) [33].

(1) $$\alpha ={\left[2^{k}\right]}^{1/4}$$

where *k* represents the number of factors.

These star points are crucial because they represent a methodology to extend beyond the range set for the experiment. Limits for this range were set at 20%–60% for LCS content and 69.3–138.7 MPa for compression pressure. Hence, a new range spreads out in space to span new concentration and pressure limits which become 12%–85% LCS (*w*/*w*) and 40.6–173.3 MPa, respectively for this work (Table 1). The new range is wide enough to specifically include the previously reported LCS content of 15% which provided the most favourable interaction with XG [32]. Although 50% *w*/*w* was the highest chitosan concentration used in previous work for controlled release preparations [28,30], the new range extends to an extreme LCS content of 85% *w*/*w* (α). The foregoing parameters were set to test whether high concentrations of the highly compactible LCS, when mixed with XG, are practically necessary in order to obtain tablets displaying a high crushing strength. The design space was then tested with a regression function whereby a quadratic equation was used to fit the response surface.

### 2.3. Powder Characterization (Micromeritic Properties)

#### True, Bulk, and Tapped Densities

The true density (*ρ*_true_) of the powders was recorded using a helium pycnometer (Ultrapycnometer 1000, Quantachrome Co., Boynton Beach, FL, USA). The cell volume was first calibrated using metal sphere standards. An appropriate amount of sample powder (2–4 ± 0.1 g) was placed in the instrument and the *ρ*_tue_ (g/cm^3^) measurements were performed, in triplicate, at a temperature of 25 ± 0.5 °C.

The bulk density (*ρ*_bulk_) of the samples was determined by weighing and pouring each powder into a 100 mL graduated glass cylinder. The volume of unsettled powder was then recorded and used to calculate the *ρ*_bulk_ (mass/volume; g/cm^3^); each sample was measured in triplicate.

Tapped density was determined by tapping a cylinder (100 mL) containing each powder using a tapping device ((SVM, Erweka GmbH, Heusenstamm, Germany) set to 100 taps. This was repeated when needed until a constant volume was attained. The new reduced volume (tapped volume) was recorded and the *ρ*_tap_ (g/cm^3^) was then calculated (as per *ρ*_bulk_) in triplicate for each sample.

### 2.4. Scanning Electron Microscopy (SEM)

The surface morphology of the studied powders and tablets produced was examined using a Hitachi SU8030 cold-cathode field emission gun scanning electron microscope (Hitachi High Technologies, Tokyo, Japan). Samples were placed on aluminum pin-type stubs after applying an appropriate adhesive. Samples were then gold coated to reduce the surface charge using a sputter coater (Edwards S150 coater). Coated samples were analyzed under high vacuum at an accelerating voltage of 10 kV. Images were previewed and collected at different magnifications using *i*-scan 2000 software (Roche Holding AG, Basel, Switzerland).

### 2.5. Compact Preparation and Characterization

Prior to mixing, XG (average particle size of 180 µm) and LCS (particle size <100 µm) powders were passed through a sieve with a mesh size of 250 μm and collected on a 90 μm mesh. Thus the particle size distribution was effectively narrowed so that LCS and XG of particle size <90 and >250 µm, respectively were discarded. Consequently, there will be almost no contribution of fine and coarse particles with diameters outside the 90–250 µm range. Raw polymers and mixtures at mass fractions of LCS of 0, 15, 20, 25, 35, 50, and 100 (*w*/*w* %) were weighed and physically mixed in a vial. A computer-controlled benchtop single punch tablet press (GTP, Gamlen^®^ Tableting Ltd. Nottingham, UK) was utilized to carry out compression experiments. Prepared samples, equivalent to 150 mg (±5 mg), were poured into a 6 mm diameter circular die. Compression was carried out using circular flat-faced punches at a speed of 60 mm/min. Five different compression forces in the range of 100–500 kg, which is equivalent to 34.6–173.2 MPa, were applied to produce compacts. At each compression pressure, three tablets were produced from each mixture in order to ensure reproducibility. The crushing force and thickness of the tablets produced were measured using a crushing forces tester (Pharma Test PTB 311E. Hainburg, Germany) and a Vernier caliper, respectively. The crushing strength was then calculated based on Equation (5) in Table 2.

### 2.6. Compression Analysis

The flowability of raw powdered polymers and their mixtures, in addition to the powder (*ε_p_*) and compact (*ε_c_*) porosity, degree of compression (DoC; %) and tablet crushing strength (σ) were analyzed using Equations (2)–(5) given in Table 2.

The in-die compressibility properties of the powders were determined by means of the Heckel and Kawakita parameters [34,35,36,37]. The Heckel equation (Equation (8)) is used to correlate the porosity reduction of powders during the compaction phase with the applied pressure *P* (MPa) as follows:

(8) $$In\;\frac{1}{1-pr}=kP+A$$

where **ρ*r* is the relative density (*ρ*_app_/*ρ*_true_) of the compacts and *k* is the slope of the linear portion of the curve that is used to calculate the yield pressure (P_y_), the inverse of *k*. P_y_ (MPa) is a measure of the plasticity of the material. The coefficient *A* indicates the extent of die filling and rearrangement of particles.

Kawakita analysis (Equation (9)) was used to evaluate compressibility via the degree of volume reduction, *C*, of powders under an applied compression pressure, *P* (MPa), as follows:

(9) $$\frac{P}{C}=\;\frac{P}{a}+\;\frac{1}{ab}$$

where *a* is a constant related to the porosity of the material, whereas the constant *b* relates to the plasticity of the material and *ab* is a constant that indicates the degree of particle rearrangement within the die. The reciprocal of *b* (1/*b*) represents the pressure required to reduce the bulk volume of the powder tested by 50%.

### 2.7. Analysis of Compression Output and Energy Consumption

**Figure 1:**
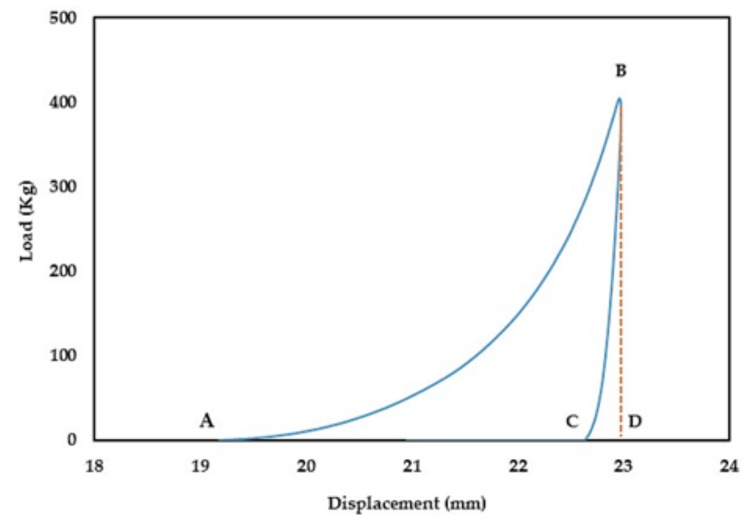
A representative GTP generated load-displacement curve of a xanthan gum (XG) sample at 400 kg load.

During the compression process (compaction and ejection), the GTP controller tracks the upper punch displacement (mm) in relation to the applied load (kg) and generates load-displacement curves. The captured data is used to estimate the work of compression (WoC) and elastic recovery (WoE) represented by areas ABD and CBD in Figure 1, respectively.

### 2.8. In-Vitro Release of Model Drugs Using the XG/LCS Matrix

Metoprolol succinate and dyphylline were chosen as two freely water soluble drugs for in vitro dissolution studies. The former drug was used to examine the relationship between the crushing strength of the tablets and the release of the drug. The latter drug was used to confirm the release phenomena at different XG/LCS ratios.

For metoprolol succinate, the procedure was carried out as follows. Metoprolol succinate (12 g) was physically mixed with 48 g of the XG/LCS matrix containing 15% *w*/*w* (7.2 g) LCS. The preparation was compressed by means of a single punch tablet press (Manesty F3 single stroke tablet press; West Pharma services Ltd., Dorset, UK) using a 9 mm circular punch. Tablet weight was adjusted to 250 mg. The samples were compressed at forces of 20, 30, 40, and 50 kN. At each compression force, 20 tablets were weighed, powdered, and then analyzed for content uniformity according to the US Pharmacopeia (USP32-NF27) for metoprolol extended release. The tablets displayed a USP compliant average assay of 99.02% (RSD = 1.9%) and an average weight of 250.9 mg (RSD = 1.5%). The average thickness of five tablets was measured using a caliper in order to determine the compact porosity. Values of crushing force of the tablets (10 tablets) resulting from the aforementioned compression forces were recorded using the crushing forces tester. Samples compressed at the aforementioned compression pressures displayed crushing forces in the following ranges: 40–80, 80–100, 100–120, and 120–150 N, respectively. These ranges are equivalent to 1.8–3.5, 3.5–4.4, 4.4–5.3, and 5.3–6.6 MPa, respectively. A USP Apparatus II with 50 rpm was set for the dissolution run. 500 mL of 0.1 M HCl was used in the first two hours of dissolution run. Keeping the tablets intact at the bottom of each vessel, the medium was discarded and replaced with 500 mL of phosphate buffer (pH 6.8). From each vessel, 5 mL of each medium was withdrawn at time intervals of 0.5, 1, 2, 4, 6, and 8 h followed by compensation with fresh media. The samples were then filtered using a 0.4 µm glass filter. Sample absorbance was measured using a UV spectrophotometer (LABINDIA UV/VIS, UV 3000, Maharashtra, India) at a wavelength of 274 nm. For each crushing strength range, the dissolution of two tablets was tested. The dissolution experiments were conducted in duplicate.

For dyphylline, formulations containing different mass fractions of LCS (0, 15, 25, 35, 50, and 100% (*w*/*w*)) were physically mixed with 100 mg of the dyphylline in order to produce tablets weighing 500 mg each. Samples were directly compressed via an industrial single punch tablet press (Manesty F3 single stroke tablet press; West Pharma services Ltd., Dorset, UK) using a 12 mm circular die with flat-faced punches. The applied force was in the range of 35–40 kN in order to maintain a similar crushing strength between all formulations. At each compression force, 20 tablets were weighed, powdered, and then analyzed for content uniformity according to the USP Pharmacopeia (USP32-NF27) for dyphylline tablets. The tablets displayed a USP compliance average assay of 98.10% (RSD = 1.9%) and an average weight of 501.3 mg (RSD = 1.5%). Dissolution studies were carried out using a USP apparatus II. Samples (10 mL) were withdrawn at time intervals of 1, 2, 4, 6, 8, 12, 16, and 20 h and replaced with fresh media. Sample aliquots were analyzed by UV spectrophotometry (LABINDIA UV/VIS, UV 3000, Maharashtra, India) at a wavelength of 273 nm.

### 2.9. Measurement of Porosity Versus Applied Pressure for Polymer Mixtures

We compressed 150 mg of binary mixtures or individual components of XG with 15% *w*/*w* of either LCS, or sodium alginate (Na ALG), or hydroxypropyl methylcellulose (HPMC) or guar gum using the GTP at compression forces of 100–500 kg using the 6 mm die. The porosity of each compact was calculated at each compression force using Equation (1). Lactose and glucosamine HCl were similarly compressed for comparison purposes.

### 2.10. XG/LCS Film Preparation and Characterization

We suspended 10 g of XG/LCS mixtures at an LCS content of 15% *w*/*w* in 500 mL of deionized water. The suspensions were spread onto Petri dishes which were kept in a vacuum oven (Vacucell, MMM Medcenter GmbH, Planegg, Germany) operating at 70 °C for 6 h. Formed films were crushed to form powder, passed over a 250 μm mesh and collected on mesh size of 90 μm. Compression was carried out by the same method described in the previous section. Compression properties and compact porosity were determined thereafter.

## 3. Results

### 3.1. Powder Characterization (Micromeritic Properties)

The data in Table 3 shows the micrometric properties of LCS, XG and their mixtures. The measured density values showed that XG powder is more dense than LCS. In relation to inter-granular porosity, LCS particles are more porous than XG particles. The Carr index (CI) and Hausner ratio (HR), Equations (6) and (7), respectively in Table 2 were calculated to evaluate the flowability of XG, LCS, and mixtures thereof according to USP 35. LCS particles showed poor flow characteristics (HR = 1.41, CI = 29.5) whilst XG particles (HR = 1.13, CI = 11.9) and XG/LCS mixture containing 15% (*w*/*w*) LCS (HR = 1.18, CI = 15.6) displayed good flowability.

### 3.2. Morphology

**Figure 2:**
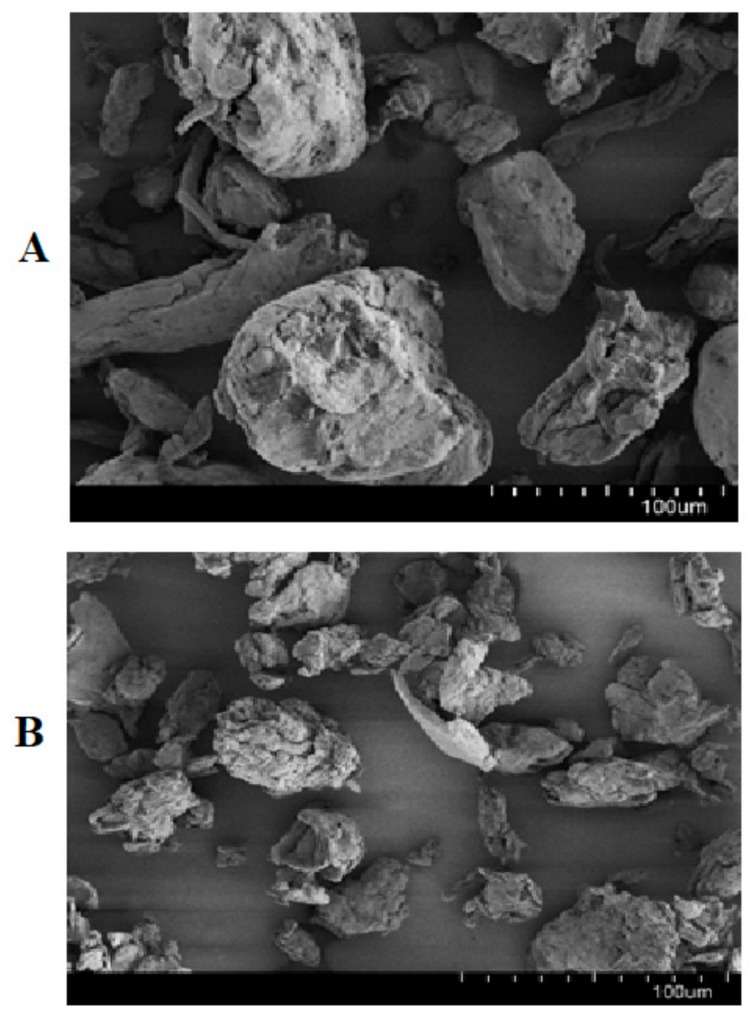
SEM images showing (**A**) XG, and (**B**) LCS powders at a magnification of 400×.

SEM was utilized to visually assess the morphology and qualitatively determine the particle size/shape. The SEM images of XG and LCS, Figure 2, show that the majority of LCS particles were smaller than 100 μm and have an average size of ~50 μm whereas the XG samples show a particle size distribution between 70 and 120 μm. As anticipated, no differences in particle shape were found; both XG and LCS consist of irregular shaped particles with rough surfaces and round shaped edges.

### 3.3. Crushing Strength Analysis

**Figure 3:**
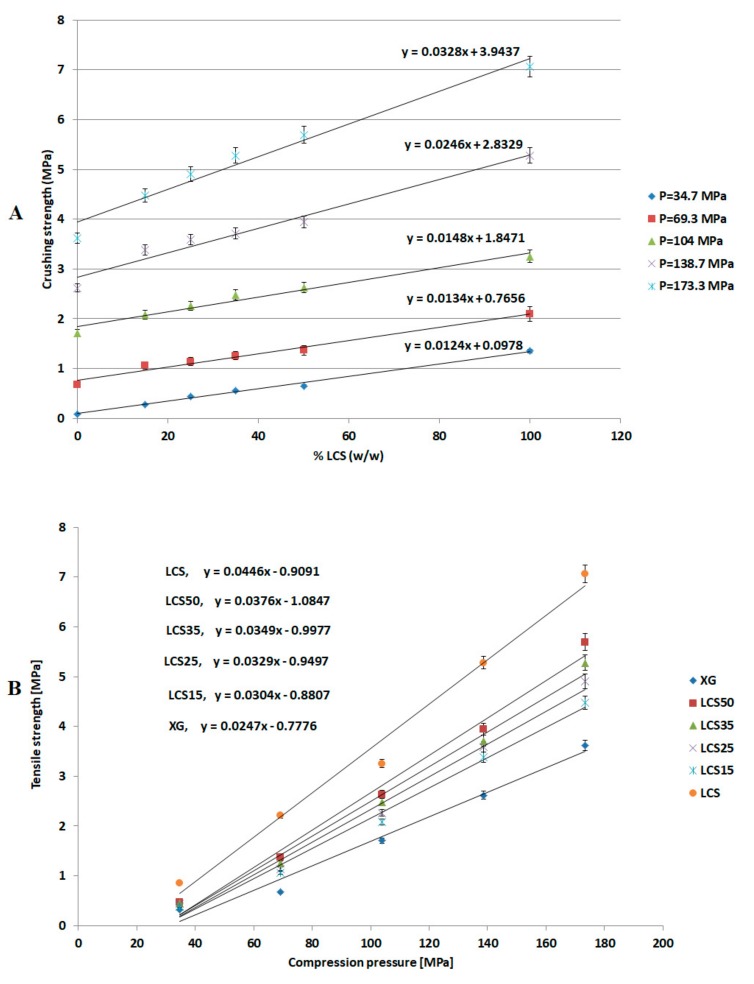
(**A**) Crushing strength versus LCS content and (**B**) crushing strength versus compression pressure.

**Figure 4:**
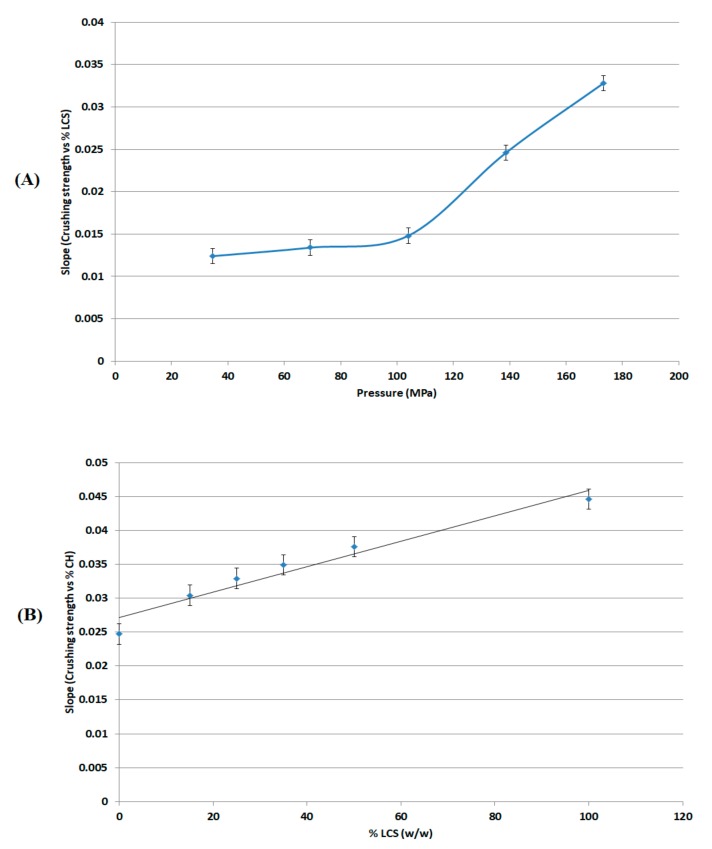
(**A**) Dependence of the change in crushing strength on the compression pressure and (**B**) dependence of the change in crushing strength on the content of LCS in the matrix.

The crushing strength (σ) values of compacts constituted of XG, LCS, and mixtures thereof produced using the GTP were measured to assess the mechanical properties (compactibility) of the tablets formed; the results are shown in Table 4. Values of σ were plotted against the LCS content in the mixture (Figure 3A) at each compression pressure; σ values were also plotted against the applied compression pressure (Figure 3B) at each LCS content. It is suggested that such plots give an indication of the sensitivity of crushing strength towards increasing compression pressure and LCS content. Initially, the data in the two figures show that LCS forms tablets with higher σ values than XG, double the σ values of XG, which indicates high compactibility. On the other hand, XG exhibits low compactibility which results in weak compacts. The aforementioned σ sensitivity was demonstrated in illustrations that describe changes in the slopes of the equations in Figure 3A,B towards changing compression pressure (Figure 4A) and LCS content (Figure 4B). In this regard, there is a clear deflection in the slope (σ versus %LCS) above compression pressure values of 104 MPa (Figure 4A). On the other hand, the slope remained constant over all the LCS concentration range (Figure 4B).

### 3.4. RSM Analysis

Coded factors, their set limits (lower to upper) and responses are presented in Table 5.

**Figure 5:**
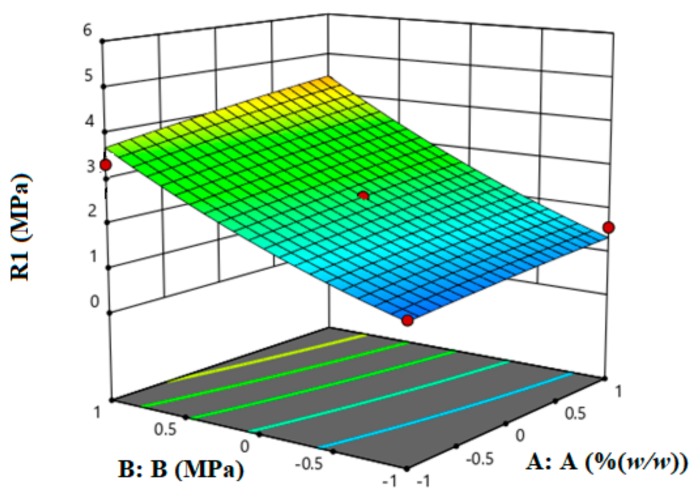
The response surface plot of the experimental crushing strength, R1 (red points) and the model predictions as a function of %LCS (A:A) and compression pressure (B:B).

The responses were best modelled using a quadratic model with the coefficient values given in Table 6. The table also shows the *p*-values for the ANOVA statistical analysis which clearly indicates the significance of the model (<0.0001). The *p*-values of the %LCS content and compression pressure factors are <0.05 which implies that the two variables are statistically significant. Moreover, when comparing the *p*-values of the two variables, it is clear that the %LCS content is less significant than the compression pressure. The experimental response (crushing strength) and the three-dimensional fitted response surface plot are shown in Figure 5. The plot represents the crushing strength (R1) as a function of %LCS (A:A) and compression pressure (B:B). The results indicate that the effect of compression pressure on the resulting crushing strength of tablets is higher than the effect of %LCS in the matrix. These results are in agreement with results presented in the previous section.

### 3.5. Compression Analysis

**Figure 6:**
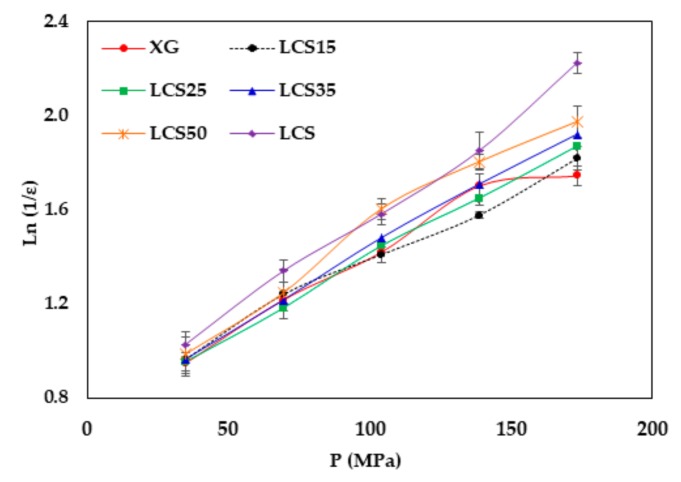
Heckel plot for XG, LCS, and their mixtures. Error bars represent SD values.

Heckel plots (Figure 6) show that porosity values (*ε_c_*) of the compacts decrease upon increasing pressure for all samples. LCS compacts displayed the lowest *ε_c_* values. The reduction in the value of *ε_c_* for XG remained constant for the compacts when the compression pressure exceeded a threshold of 138 MPa. On the other hand, a considerable reduction in *ε* for LCS and, to a lower extent, XG/LCS compacts was still obtained indicating that LCS is more compressible than XG and, more importantly, it can modify the compression behaviour of XG.

In order to understand the deformation mechanism of the studied powders, the yield pressure (P_Y_) was calculated from the linear portion of the Heckel plot. Since P_Y_ is the inverse of the slope (k) of the linear portion of the plot and is related to the plastic deformation ability of materials, low P_Y_ values indicate high plasticity of materials. The results shown in Table 7 suggest that LCS deformation is governed mainly by a plastic deformation mechanism. Whereas, XG showed a low tendency to plastic deformation which suggests the dominancy of brittle fracture behaviour; whereby particles undergo fragmentation under applied pressure. XG/LCS mixtures exhibited P_Y_ values in the range of those exhibited by the two polymers used, which indicates the occurrence of multiple deformation processes.

**Figure 7:**
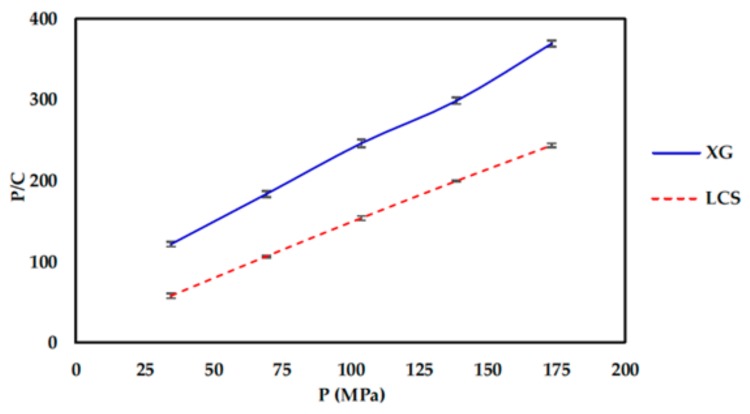
Kawakita plots for XG and LCS.

Kawakita plots for XG and LCS compacts are presented in Figure 7. At the same compression pressure values, LCS compacts display a higher degree of volume reduction than XG. This explains the more dense tablets with higher σ values resulting from LCS samples and indicates, once more, the high compressibility of LCS. In contrast, XG displayed a much lower volume reduction during compression. The parameters *a*, 1/*b*, and *ab* are presented in Table 7. Referring to the calculated values, LCS exhibits high compressibility and volume reduction, whereas XG exhibited the lowest value for the “*a*” parameter. The extent of plastic deformation of materials can be estimated from the Kawakita parameter “1/*b*”. XG showed the highest “1/*b*” value exceeding the value of LCS by four-fold. This further indicates the brittle/fracture nature of XG particles under pressure. The plastic deformation behaviour of mixtures is dependent on the mass fraction of LCS such that “1/*b*” decreases with the fraction of LCS. The foregoing is another manifestation of the predominant contribution of LCS in the LCS/XG matrix. *ab* values (Table 7) indicate the increasing extent of particle rearrangement with LCS mass fraction, reaching a maximum when LCS is used alone.

**Figure 8:**
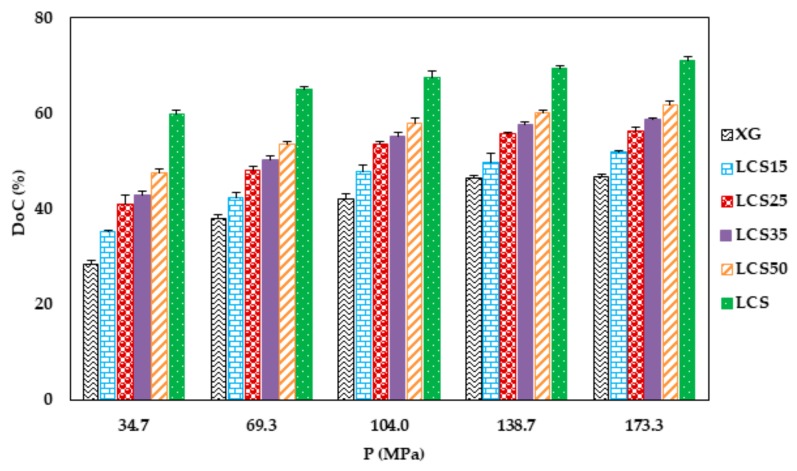
Degree of compression for XG, LCS and their mixtures. Error bars represent SD values.

Data for the degree of compression (DoC) for the compacts produced are presented in Figure 8. The results demonstrate the high compressibility and extent of packing of LCS. However, XG showed a low extent of packing; it was almost half the value of LCS at low compaction pressures, which remained fairly constant at high pressures (138 and 173 MPa). This correlates well with the results from the Heckel plot in which a plateau was reached at compaction pressures ≥138 MPa. Mixtures of XG and LCS demonstrate DoC values in the range of the two polymers which kept increasing even for mixtures with a low fraction of LCS.

### 3.6. Force-Displacement Curve Analysis

**Figure 9:**
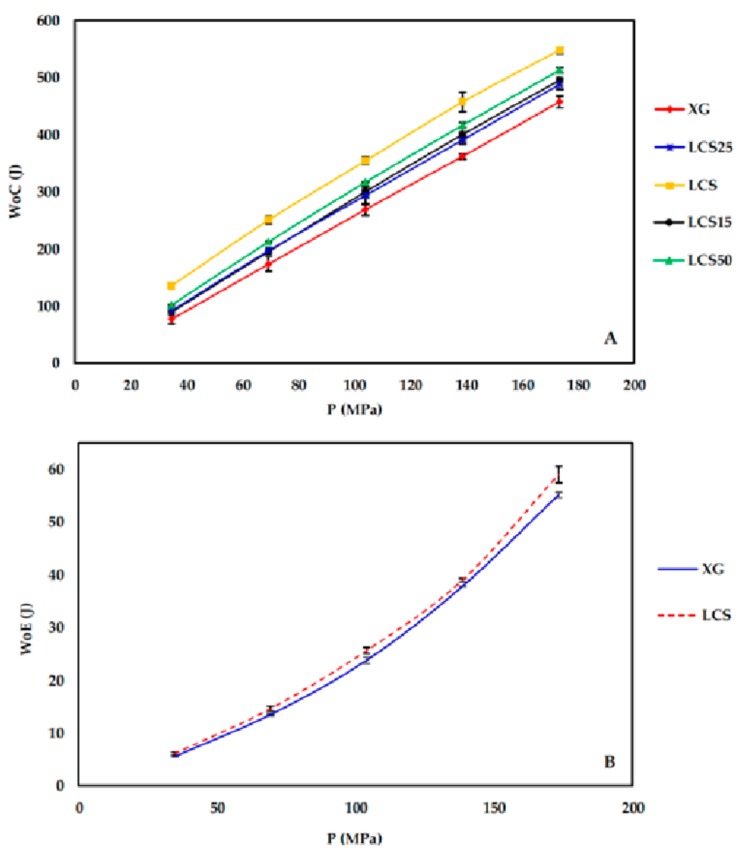
(**A**) Work of compression (WoC) and (**B**) work of elastic recovery (WoE) for XG and LCS. Error bars represent SD values.

The data presented in Figure 9A,B display the work of compression (WoC) and elastic recovery (WoE), respectively, as a function of applied compression pressure. Both parameters were calculated from the load-displacement curves. It is evident that the WoC increases with pressure for all samples. WoC values increased in the presence of LCS, whilst XG exhibited the lowest WoC. High stored energy within the tablets, due to compression, can be recovered as elastic work at the decompression stage. Calculated WoE values showed that XG and LCS released almost the same levels of energy (Figure 9B). WoE values for XG/LCS mixtures were not plotted, for clarity, since their values are very similar to XG and LCS. It is to be noted that LCS stored high energy in the compression phase and released a small fraction of it upon decompression. This could explain the high crushing strength of LCS compacts. On the other hand, XG compacts displayed lower WoC values whereas no significant difference in the WoE between XG and LCS compacts was detected.

### 3.7. Effect of LCS and Different Excipients on Porosity Reduction of Xanthan Gum

**Figure 10:**
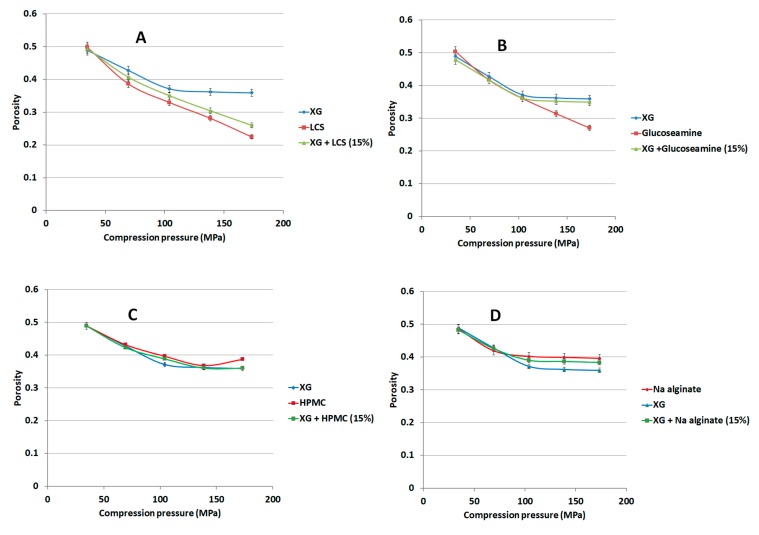
Dependence of matrix porosity on its composition and compression pressure for XG/LCS (**A**), XG/glucosamine (**B**), XG/HPMC (**C**), and XG/ALG (**D**). Hydroxypropyl methylcellulose (HPMC), Na alginate (ALG).

**Figure 11:**
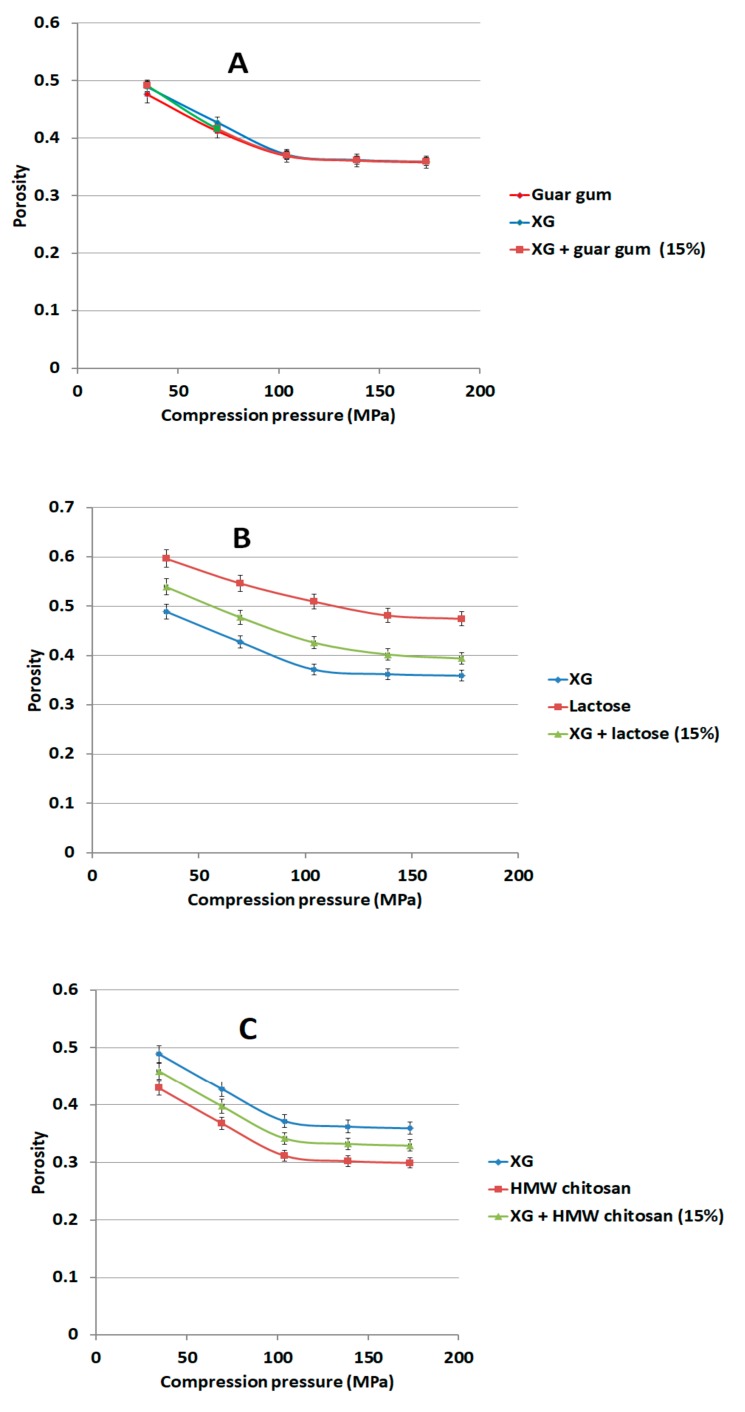
Dependence of matrix porosity on its composition and compression pressure for XG/guar gum (**A**), XG/lactose (**B**) and XG/high molecular weight (HMW) chitosan (**C**).

The choice of LCS was investigated in relation to its contribution to a reduction in porosity when a compression pressure is applied to XG powder. The reduction in the porosity of XG was found to be independent of the applied compression pressure when the value of the latter starts to exceed 138.7 MPa (Figure 10 and Figure 11). Polymers commonly utilized in controlled release studies such as glucosamine, HPMC, Na ALG, guar gum, lactose, and high molecular weight (HMW) chitosan were added to XG to compare their effect on XG porosity. Glucosamine and lactose were also used because the former is a constituent of the structure of the chitosan polymer, whilst lactose is widely used as a filler in tablets. When 15% *w*/*w* LCS was present with XG, porosity continued to decrease with applied pressure even at values higher than 138.7 MPa (Figure 10A). Such a decrease is typical of the behaviour of LCS when it is compressed as a single excipient. In fact, this behaviour was found to be a unique characteristic of LCS compared to other common excipients. In this regard, binary mixtures of XG with either glucosamine, HPMC, Na ALG, guar gum, lactose, or high molecular weight (HMW) chitosan displayed a porosity-pressure profile similar to that of XG (Figure 10 and Figure 11). In other words, the binary mixtures of XG and the aforementioned excipients, except LCS, were found to be incompressible after a specific compression pressure. This property makes the XG/LCS mixture suitable for compression into compacts at different crushing strengths and also potentially allows a wide spectrum of APIs to be used at a certain compression force which modifies their release behaviour.

**Figure 12:**
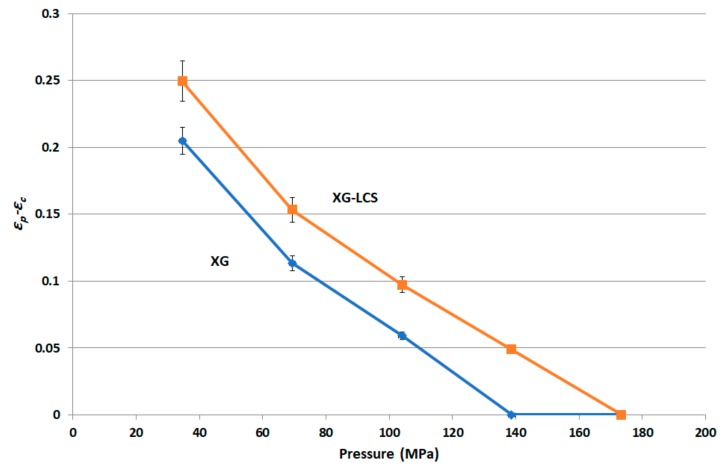
Porosity versus compression pressure plots for XG/LCS matrix and XG.

In order to detect any percolation behaviour in the relationship between porosity and the applied pressure, the difference between calculated powder porosity (*ε_p_*) and the minimum compact porosity value (*ε_c_*) attained as a function of applied pressure was plotted for the XG/LCS mixture at LCS content of 15% *w*/*w* (Figure 12).

### 3.8. In-Vitro Release of Metoprolol Succinate and Dyphylline

**Figure 13:**
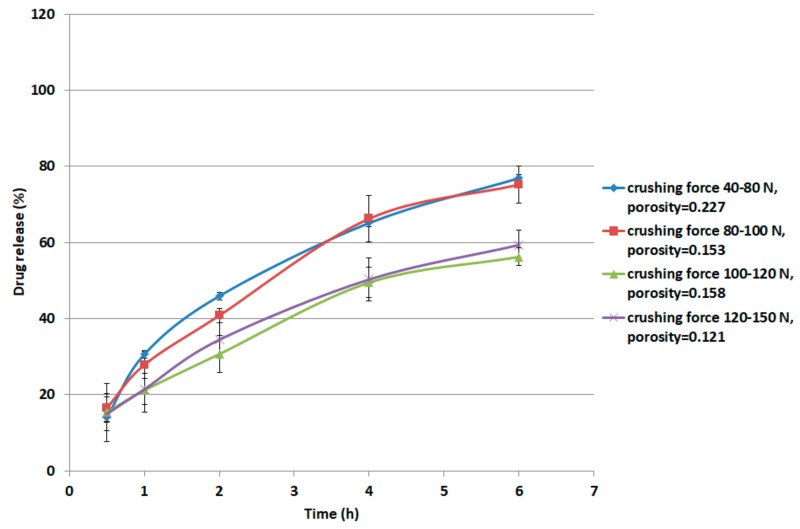
Relationship between metoprolol succinate release from XG/LCS matrix and its crushing strength and porosity.

The relation between the crushing strength and drug release of tablets comprising 50 mg metoprolol succinate as an active pharmaceutical ingredient and 200 mg XG/LCS as the controlled release matrix is presented in Figure 13. The four ranges of crushing strength show controlled release behaviour within 6 h of dissolution. Drug release at the first two low crushing strength ranges (1.8–3.5 MPa and 3.5–4.4 MPa), was greater than that at the higher crushing strength ranges used (4.4–5.3 MPa and 5.3–6.6 MPa). In addition, drug release of the first two ranges showed almost the same dissolution profile. However, the foregoing presented release profiles with a wider difference than that of the two matching high crushing strength ranges. Such observation was clear starting from the second hour up to the sixth hour of dissolution. This duration was enough to differentiate between compacts having variations in crushing strength. On the other hand, compacts porosities were higher for tablets of high crushing strength, as indicated in the legend in Figure 13.

**Figure 14:**
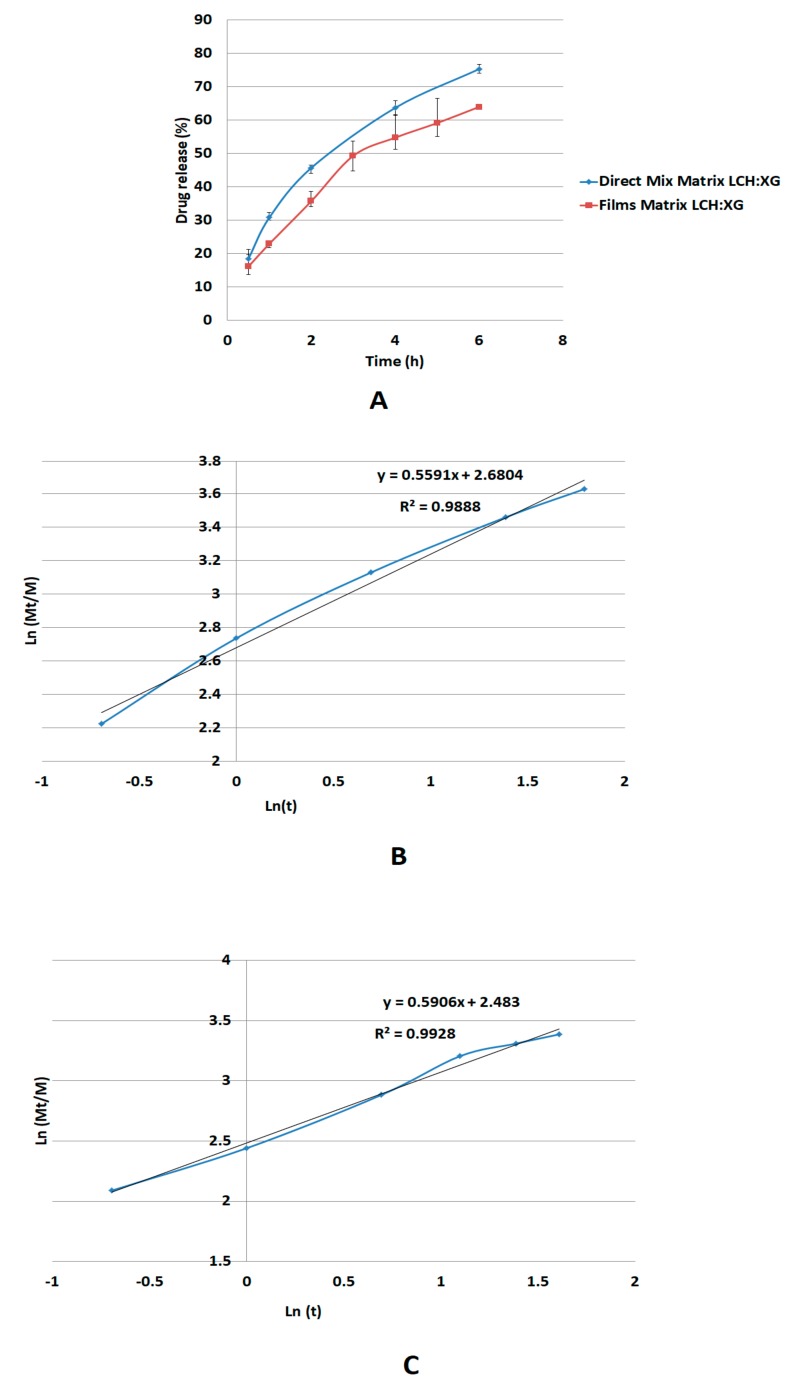
Profiles for metoprolol succinate using XG/LCS matrix as a physical mixture or as a dried gel powder of the same composition (**A**). Regression analyses, Ln (Mt/M) versus Ln time, for metoprolol succinate physical mixture (**B**) and dried gel matrices (**C**). (Mt/M is the fraction of drug released at time t.

The release mechanism of metoprolol matrix comprising XG/LCS was investigated against a reference matrix comprising a dried gel powder (film) of the same composition XG/LCS ratio. The aim of such a reference was to highlight the impact of the physical mixture undergoing gelling on drug diffusion when compared to an already gelled phase. Results of drug dissolution in the aforementioned systems are presented in Figure 14.

**Figure 15:**
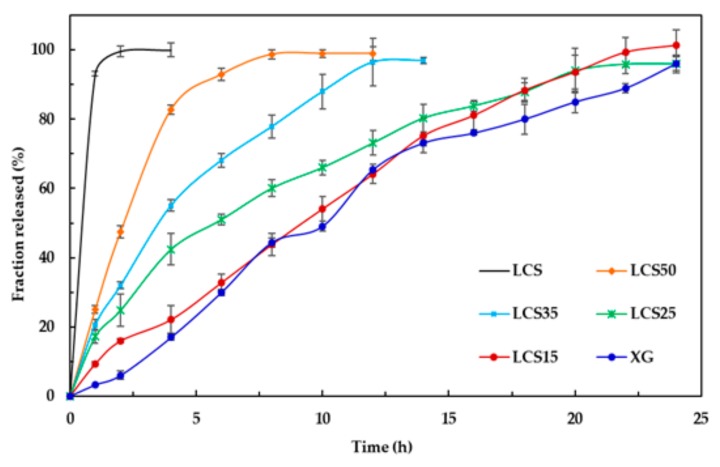
In-vitro release profiles for dyphylline from XG/LCS compacts at different mass percentages of LCS. Error bars represent SD values.

Dyphylline was chosen as another model drug to assess the efficiency of the LCS-XG based compacts as controlled-release drug carriers. This is due to its free solubility in water (>333 mg/mL) and short biological half-life (~2 h) [38]. The in-vitro release profiles for dyphylline from XG, LCS and their mixtures are shown in Figure 15. Dyphylline shows immediate release from LCS compacts, while its release was hindered when incorporated in XG based matrices. Using both XG and LCS causes a prolongation in the API release of up to 6–8 h for the 50% LCS tablet mixture. Reducing the mass fraction of LCS results in further drug retardation capability of the tablets. Hence, XG/LCS mixtures can be used to modify the release of dyphylline.

## 4. Discussion

This work aimed to assess the contribution of LCS on the performance of XG in pharmaceutical manufacturing and processing of controlled release solid dosage forms. From a powder flow point of view, the good flow properties of XG as indicated by the measured Carr index and Hausner ratios (Table 3), compared to LCS, necessitates the use of lower concentrations of LCS whenever a combination of the two excipients is desired. Despite similarities in particle size and shape of the two excipients, the apparent improvement in powder flow for mixtures of high XG content is attributed to the higher bulk density of XG compared to that of LCS (Table 3). Accordingly, the low porosity recorded for XG when compared to LCS is obviously a result of the highly dense nature of XG. This makes the combinations of these polymers a practical prerequisite to obtain a DC matrix.

The most effective added value that LCS imparts to the XG/LCS mixture is the remarkable increase in the crushing strength of the fragile XG compacts. The extent of such an increase was found to be sensitive towards applied compression pressure rather than increasing the LCS content in the mixture (Figure 3). This was confirmed by the sudden deflection, from almost a plateau state, in the slope of the profile above a compression pressure of 104 MPa whereas the slope remained constant at all % content of LCS (Figure 4). In other words, hard compacts can be predominantly obtained above specific applied pressure values and upon increasing LCS content. Optimal responses using RSM procedure confirmed the low attribute of LCS content in tablets crushing strength. To be more specific, the RSM technique indicated the low impact of LCS content to achieve hard compacts especially when high compression pressures (>104 MPa) were used. Accordingly, an LCS content of 15% (*w*/*w*) in the mixture represented an optimum value at the minimum chitosan concentration that can produce hard compacts when high compression pressures are used. Moreover, the use of LCS at an optimum fractional content of 15% (*w*/*w*) supports a previous finding on optimum LCS content for interaction between XG and LCS using molecular dynamics simulations. The later study confirms that the most favourable complex is formed at an LCS content of 15% *w*/*w* [32]. On the other hand, the use of LCS at low concentrations within the XG/LCS mixture is advantageous when compared to reported work on high molecular weight chitosan and XG mixtures [27,28]. The later mixtures showed optimum controlled release behaviour when high chitosan content was used or more specifically at the XG: high molecular weight chitosan mass ratio of 1:1.

Irrespective of the content of LCS, its presence with XG is practically imperative from a powder compression perspective. Its main significance in the mixture was found to be attributed to the high extent of plastic deformation of LCS as illustrated in Heckel (*P_Y_*) and Kawakita (1/*b*) analysis upon compression of the powder. The last technique further emphasized the importance of having LCS in the mixture to overcome low powder compressibility (*a*) generally attained by XG. This was evident in the high particle rearrangement (*ab*), high volume reduction (*C*), and high degree of compression (DoC) values of the powders when LCS was included in the mixture.

Since materials with high compression work values utilize the input energy for deformation, accordingly the high compression work displayed by the mixture is attributed to LCS as a highly deforming material [39]. Theoretically, the compression work is a product of compression force times the magnitude of punch displacement; this implies that the high energy displayed upon compression of the mixture is attributed to LCS as a highly compressible material.

The rigid, low porous structure of XG was further found to lose its compressibility around a percolation pressure threshold (i.e., >104 MPa) above which no change in the compact porosity was attained. On the other hand, the inclusion of LCS, at low content, i.e., 15% *w*/*w*, was found to eliminate the pressure-porosity threshold as a further reduction in powder porosity was recorded above 104 MPa. Bearing in mind that this value—as discussed earlier—represents the compression pressure above which high tablet crushing strength can be obtained, it is suggested that there exists a combination of two possible explanations for such hard compacts. The first is due to the predominant effect of high compression pressures (>104 MPa) in eliminating the weak ability of XG to form hard compacts on its own. The second is related to the unique behaviour of LCS in enhancing powder volume reduction and further porosity reduction which are the two main factors for improved compressibility of XG (Figure 10A). In fact, continuous reduction in porosity did not take place above pressures of 104 MPa when XG was mixed with other additives and copolymers such as; controlled release excipients (Na ALG, HPMC, and guar gum), fillers (lactose), the unit structure of LCS material, i.e., d-glucosamine HCl, and the high molecular weight chitosan (HMW chitosan) (Figure 10 and Figure 11). Results confirm a previous finding on the higher extent of powder packing from the initial powder volume for LCS when compared to HMW chitosan [31]. The difference in powder packing was suggested to be related to the structural configuration difference, from intermingled molecular to open structure, as the MW is decreased. Moreover, it was found that differences in helical packing contribute to the transfer from plastic to brittle-fracture nature with the high extent of packing of chitosan upon compression when the MW is reduced [31]. Based on the foregoing, LCS undergoes a higher reduction in powder porosity than HMC confirming the results shown in Figure 10A and Figure 11C, respectively.

It is believed that changes in XG/LCS compact porosity with compression pressure are directly related to tablet crushing strength; the latter was found to be pressure sensitive rather than influenced by LCS content. These changes were found to be valid whether around a percolation threshold (for XG) or in a decreasing behaviour (for the XG/LCS mixture), both taking place upon increasing the compression pressure. In other words, the elimination of porosity percolation threshold of XG by the inclusion of LCS did not deter the presence of two different dissolution profiles ranges of metoprolol tablets which were found to be dependent on tablet crushing strength. The foregoing was recorded above a tablet crushing strength of 4.4 MPa when metoprolol drug release remained unchanged irrespective of tablet crushing strength. Below this limit, drug release was faster for the two crushing strength ranges (1.8–3.5 MPa) and (3.5–4.4 MPa) of the tablets. The existence of these two dissolution profile ranges reflects the sensitivity of compact crushing strength to compression pressure values as earlier discussed in relation to the data presented in Figure 4A. Such drug release, pressure or crushing strength dependence, excluded the possibility of any contribution of porosity percolation threshold behaviour as the values of measured porosity for the tablets were in decreasing order as illustrated in Figure 10, Figure 11 and Figure 12. In this regard, the LCS/XG tablets encountered a continuous decrease in porosity with an increase in tablet crushing strength. This further emphasizes the fact that LCS modifies drug release by eliminating the porosity/pressure percolation threshold encountered with XG; however, tablet crushing strength played a dominant factor in drug release. The latter behaviour provides another opportunity to modify the extent of drug release via the adjustment of tablet crushing strength without the need to add release modifying excipients.

The release mechanism of metoprolol succinate through the XG/LCS gel layer follows non-Fickian diffusion suggesting anomalous transport of the drug rather than through erosion of the matrix [40,41,42]. Apparently, the calculated *n*-value of the Korsmeyer-Peppas model at a value of 0.59, which lies between values of 0.45 and 0.89, justifies this type of diffusion [42]. Moreover, the mechanism of diffusion through a gel phase formed from XG/LCS physical mixture is unexpectedly similar to that made up from a dried film of the gelled mixture of *n*-value = 0.55. Although the latter value reflects non Fickian diffusion, it is still lower than that of the physical mixture and approaching the value of the Fickian behavior (*n* = 0.45). This indicates that that the gel structure from a dried film participates in partial regulation of drug release presumably via the build-up of a highly porous matrix structure.

On the other hand, drug release is faster in an acidic medium compared to a basic medium. The foregoing can be explained on the basis of the dependence of the solubility on pH for metoprolol succinate which decreases with increasing pH [43]. Such a decrease is attributed to the chemical structure of metoprolol succinate salt, which is made of the metoprolol cation and the dicarboxylate anion in a 2:1 ratio [44] with a pKa of 9.67 [45]. In this regard, this compound undergoes protonation in the acidic pH range, at the amine moiety, leading to enhanced drug solubility. On the other hand, when the pH is increased, the compound becomes less protonated until a free base is formed causing a reduction in its solubility [46].

Finally, LCS does not provide an extended release effect, which was predominantly presented by XG, when their influence was tested on dyphylline. In fact, the presence of LCS at the content of 15% *w*/*w* provides the extended release profile of dyphylline for up to 12 h of dissolution time. The release profile of the drug with XG was slower compared to that for the matrix system. Therefore, the inclusion of LCS at low content (15% *w*/*w*) within the XG/LCS matrix can maintain intact compacts without any disintegration while extending drug release profiles above 12 h of dissolution time. Thus mixtures of XG/LCS present a unique combination in forming intact matrices that modulate extended release of freely soluble drugs by varying compression force.

Examination of the drug release profiles in acidic and basic media for all the matrices comprising dyphylline (Figure 15) show that—similar to metoprolol succinate—the release of the drug slows down when the medium changes from an acidic to a basic one. Once again, the chemical structure of dyphylline plays the main role in such release behavior. In this regard, the amphoteric nature of dyphylline with two pKa values (−1 and 8.5–10) allows protonation of the basic group at pH values <2 and >8.5 [45,47,48]. This gives rise to increased drug solubility in the HCl dissolution medium (pH 1.0). In contrast, drug solubility encounters a decrease in the phosphate buffer (pH = 6.8) as its remains deprotonated.

## 5. Conclusions

Results confirmed the contribution of LCS to enhanced powder compressibility and high compacts crushing strength. Compactibility of the XG/LCS mixtures was found to be more sensitive to applied compression pressure than to LCS content. This allows the mixture to be adaptable to a wide range of compression forces due to the ability of LCS to reduce powder porosity when mixed with XG. Low LCS content was found to maintain a DC matrix. RSM analysis showed that the optimum content of LCS in the LCS/XG matrix was around 15%. Furthermore, the matrix which kept its high integrity up to 24 h can be used to control the drug release of different APIs. The reported system is achievable by using conventional powder processing techniques with satisfactory powder flow behaviour.

## Figures and Tables

**Table 1:** Design of experimental factors and their corresponding codes. Low molecular weight chitosan (LCS).

Codes	Factor 1 (LCS Content, % *w*/*w*)	Factor 2 (Compression Pressure, MPa)
−α	12	40.6 (117 kg)
−1	20	69.3 (200 kg)
0	40	104 (300 kg)
1	60	138.7 (400 kg)
+α	85	173.3 (500 kg)

**Table 2:** Equations used for the calculation of tableting parameters.

Property	Equation *	Number
Powder porosity ($\varepsilon_{p}$)	$\left(1\;-\;\frac{\rho_{bulk}}{\rho_{true}}\right)$	(2)
Compact porosity ($\varepsilon_{c}$)	$\left(1\;-\;\frac{\rho_{app}}{\rho_{true}}\right)$	(3)
Degree of compression (DOC)	$\left(\frac{H_{0}\;-\;H_{p}}{H_{0}}\right)\;\times \;100$	(4)
Crushing strength (σ)	2F/πDh	(5)
Carr index	$\left(\frac{\rho_{tap}\;-\;\rho_{bulk}}{\rho_{tap}}\right)\;\times \;100$	(6)
Hausner ratio	$\left(\frac{\rho_{tap}}{\rho_{bulk}}\right)$	(7)

* *ρ*_bulk,_ *ρ*_tap,_ represent bulk, tapped, and true densities. The apparent density (_app_) of the compacts was calculated from the weight (g) and volume (cm^3^) of the compacts. The degree of compression (DoC; %) gives an indication of the extent of packing of a powder under compression whereby *H*_0_ and *H_p_* are the height (mm) of the filled powder before and after compression, respectively. For the crushing strength (σ, MPa) of the produced compacts; *F* is crushing force (N), *D* is tablet diameter (m), and *h* is tablet thickness (m).

**Table 3:** Micrometric properties of XG, LCS, and their mixtures ^a^.

Sample	*ρ*_true_^b^	*ρ*_bulk_^b^	*ρ*_tapped_^b^	Porosity	Carr Index	Hausner Ratio
**XG**	1.52 (0.01)	0.66 (0.00)	0.77 (0.01)	0.56 (0.00)	11.9 (0.31)	1.13 (0.01)
**LCS**	1.43 (0.03)	0.37 (0.01)	0.52 (0.00)	0.74 (0.02)	29.5 (1.09)	1.41 (0.02)
**LCS50**	1.47 (0.01)	0.48 (0.01)	0.62 (0.01)	0.68 (0.01)	22.5 (1.06)	1.29 (0.03)
**LCS35**	1.49 (0.01)	0.52 (0.01)	0.66 (0.00)	0.65 (0.01)	21.2 (0.93)	1.27 (0.02)
**LCS25**	1.50 (0.00)	0.56 (0.01)	0.69 (0.01)	0.63 (0.01)	17.5 (1.33)	1.22 (0.02)
**LCS15**	1.51 (0.01)	0.61 (0.00)	0.71 (0.01)	0.60 (0.00)	15.6 (0.81)	1.18 (0.00)

^a^ Numbers in parentheses represent SD from the average values. ^b^ Density values are in g/cm^3^.

**Table 4:** Values of σ (MPa) for XG/LCS mixtures compressed at a pressure range of 34.7–173.3 MPa *.

Sample	Pressure (MPa)				
	34.7	69.3	104.0	138.7	173.3
**XG**	0.32	0.67	1.71	2.62	3.62
**LCS50**	0.47 (0.01)	1.37 (0.01)	2.63 (0.06)	3.95 (0.10)	5.69 (0.12)
**LCS35**	0.45 (0.06)	1.26 (0.05)	2.48 (0.03)	3.71 (0.08)	5.28 (0.10)
**LCS25**	0.44 (0.08)	1.14 (0.04)	2.26 (0.06)	3.59 (0.10)	4.91 (0.12)
**LCS15**	0.38 (0.01)	1.06 (0.01)	2.08 (0.08)	3.38 (0.02)	4.48 (0.11)
**LCS**	0.86	2.21	3.254	5.28	7.06

* Numbers in parentheses represent the SD from the average values.

**Table 5:** Response surface methodology (RSM) parameters for coded factors and their responses for the XG/LCS system.

	Factor 1 (% *w*/*w*)	Factor 2 (MPa)	R1 (Crushing Strength, MPa)
1	1	−1	1.57
2	1	1	4.31
3	0	0	2.44
4	−α	0	2.19
5	−1	1	3.32
6	0	0	2.44
7	0	α	5.26
8	0	0	2.44
9	α	0	2.68
10	0	0	2.44
11	0	0	2.44
12	0	−α	0.59
13	−1	−1	1.03

**Table 6:** ANOVA analysis for the crushing strength of XG/LCS as a function of LCS content and compression pressure.

Source/Factors	R1	
	Coefficient Estimate of the Actual Equation	*p*-Value
Model *		<0.0001 Significant
Intercept	2.44	
A	0.276	0.0154
B	1.45	<0.0001
AB	0.112	0.3916
A^2^	−0.031	0.7513
B^2^	0.212	0.0566

* Equation: *Y* = 2.44 + 0.276A + 1.45B + 0.112AB − 0.031A^2^ + 0.212B^2^, where Y: R1 (MPa), A: LCS content (% *w*/*w*), B: compression pressure (MPa).

**Table 7:** Calculated compressibility parameters for XG, LCS, and their mixtures.

Sample	P_Y_ (MPa)	*a*	1/*b*	*ab*
**XG**	213.48 (8.4)	0.57 (0.01)	34.42 (1.19)	0.02
**LCS**	97.340 (2.9)	0.77 (0.03)	8.910 (0.11)	0.09
**LCS50**	125.71 (4.1)	0.67 (0.02)	16.01 (0.06)	0.04
**LCS35**	128.22 (3.5)	0.64 (0.01)	17.89 (0.33)	0.03
**LCS25**	133.45 (5.7)	0.63 (0.00)	19.11 (0.90)	0.03
**LCS15**	137.20 (4.5)	0.60 (0.02)	25.46 (0.04)	0.02

* Numbers in parentheses represent SD values.

## Chemical Compounds Studied in This Article

Chitosan (PubChem CID: 21896651); dyphylline (PubChem CID: 3182); metoprolol succinate (PubChem CID: 62937); xanthan gum (PubChem CID: 7107).

## Abbreviations

APIs: active pharmaceutical ingredients; DC, direct compression; DoC, degree of compression; Gamlen tablet press, GTP; LCS, low molecular weight chitosan; RSM, response surface method; GTP WoC, work of compression; WoE, work of elastic recovery; XG, xanthan gum.
